# Experiences of returning to work after sick leave due to exhaustion disorder: a qualitative content analysis

**DOI:** 10.1186/s40359-025-03873-9

**Published:** 2025-12-20

**Authors:** Sanna Forslund, Filip Jovicic, Annika Näslund, Helen Habte, Jakob Clason van de Leur, Monica Buhrman, Alexander Rozental

**Affiliations:** 1 Department of Psychology, Uppsala University, Uppsala, Sweden; 2 Department of Health, Education and Technology, Luleå University of Technology, Luleå, Sweden; 3 Department of Clinical Neuroscience, Karolinska institutet, Stockholm, Sweden

**Keywords:** Stress, Exhaustion disorder, Return to work, Qualitative content analysis

## Abstract

**Background:**

Non-traumatic stress-related illnesses are associated with considerable functional impairment and costs for both individuals and societies. In Sweden, the national diagnose Exhaustion disorder (ED) is one of the most frequently diagnosed disorders in this category. Despite the major negative consequences of illnesses such as ED, there is no clear evidence on successful treatments or effective interventions to enhance return to work outcomes for this population. Therefore, the aim of this study was to explore how individuals who had returned to work after sick leave due to ED experienced the process of returning to work, including facilitating factors, significant challenges and needs of further support.

**Methods:**

Participants were recruited from three clinics in Stockholm specialized in rehabilitation for Exhaustion Disorder. Semi-structured interviews were conducted with 15 participants and the transcribed interviews were analyzed using qualitative content analysis.

**Results:**

The results yielded three themes – Struggling to adapt sustainably to change, Being supported or hindered by the context and Being part of a larger societal system. The first theme revolves around individual processes of change, such as gaining insight in one’s own behavior, and rethinking one’s perceived identity. The second theme focuses on how practical and emotional support, or lack thereof, affect the experience of returning to work. The third theme addresses hindering or facilitating factors on a more societal level, such as social norms or conditions of the social welfare system.

**Conclusions:**

The results indicate the relevance of further and prolonged support for individuals returning to work after sick leave due to ED. The workplace and overall organization plays an important role in providing this support. In a clinical individual context, the support given may include programs designed to facilitate behavioral maintenance, sustainable psychological flexibility and renegotiation of values. However, further research is needed on how such programs should be best designed and delivered.

**Trial registration:**

Not applicable.

**Supplementary Information:**

The online version contains supplementary material available at 10.1186/s40359-025-03873-9.

## Background

Stress-related illnesses are one of the most common mental health problems in many European countries [1, 2]. The conceptualization of non-traumatic stress-related illnesses differs between countries, and some examples of concepts used are chronic burnout, clinical burnout, stress-related exhaustion and job stress-related depression [3]. Regardless of conceptualization, non-traumatic stress-related illnesses are associated with everyday functional impairment and the costs and losses due to sick leave and reduced productivity are substantial for both individuals and society [4–6].

In Sweden, stress-related disorders represent 50% of all initiated sick leaves due to mental health problems [7]. The Swedish diagnosis stress-induced exhaustion disorder (ED) [F43.8 A Utmattningssyndrom] is the diagnosis most commonly used in this category. ED was introduced to the Swedish version of the International Statistical Classification of Diseases and Related Health Problems, 10th Revision (ICD-10-SE) in 2005 and has been discussed as a more severe clinical manifestation of the occupational phenomenon burnout [8]. Burnout itself is an occupational phenomenon included in the 11th Revision of the International Classification of Diseases (ICD-11). The core criteria for ED are defined as mental and physical exhaustion and the onset must be preceded by identifiable stressors for at least six months. Stressors are often work-related, such as high work demands, but can also manifest themselves in private life as relational problems or caring for a family member [9].

Due to the great distress and debilitation that characterizes stress-related disorders such as ED, different treatments have been proposed. Cognitive Behavior Therapy (CBT) and multimodal rehabilitation (i.e., interdisciplinary interventions delivered by teams of physicians, psychologists, physiotherapists, and occupational therapists) have shown some promising results for symptom recovery [10–14]. However, results do not show strong evidence in favor for any particular treatment [15]. In addition to studies on symptom reduction, a recent systematic review and meta-synthesis of qualitative studies highlights what factors are experienced as being helpful for the recovery process from stress-related disorders [16]. The results show that recovery is experienced as a dynamic process where a supportive environment that foster safety as well as openness to several perspectives of personal growth are key elements for meaningful change.

Knowledge on how to promote Return-To-Work (RTW) for this population is also limited. A few studies have shown encouraging results for RTW-interventions specifically targeting ED. One study found that a workplace-oriented intervention showed a long-term stability effect on RTW [17], but only for participants 45 years or younger. Another study showed a small and diagnostic specific effect of a workplace intervention on sick absence in participants with ED [13]. In general, studies on RTW interventions for burnout or common mental disorders (CMD) have yielded mixed results. A review of RTW interventions for employees on sick leave due to burnout [18] found that only one of eight included studies showed a significant effect on RTW. Further, the authors state that no conclusions can be drawn from the results due to high heterogeneity and risk of bias in the studies included. This is in line with research on RTW for CMD, where multiple studies [19–23] have tested different interventions for increasing RTW without any significant positive results for the effect of the interventions. A systematic review [24] concluded that work-focused CBT and work-focused team-based support showed a slight increase in RTW for individuals with CMD, but that there is low certainty for the evidence. Hence, there is no consensus in the field on what promotes RTW in this population.

RTW for CMD has also been explored from a qualitative perspective. A meta synthesis of qualitative studies [25] showed that implementations of RTW interventions or strategies for individuals with CMD were not always successful, and that unsuccessful implementation could lead to relapse and recurring sick leave. The authors found that interventions based on the biopsychosocial model, where both biological factors (i.e., symptoms like exhaustion and forgetfulness), psychological factors (i.e., perfectionism, low self-efficacy) and social factors (i.e., lack of coordination between stakeholders) are taken into account seemed to be more successful than others. The authors called for future qualitative studies on the experience of RTW from the perspectives of people with CMD, in order to explore facilitating factors and prevent relapse in sick leave.

Approximately 50% of individuals diagnosed with ED still report fatigue and decreased stress-tolerance up to seven years after first seeking and receiving treatment [26]. Reduced cognitive abilities have also been found several years later [27, 28]. This could be a contributing factor to the high risk of relapse in sick leave in individuals with ED [3, 27, 28]. A report by the Swedish Social Insurance Agency [29] shows that approximately one third of patients previously on sick leave due to mental disorders relapse into sick leave again within three years. This number applies to all mental disorders, but as mentioned above, stress-related disorders represent 50% of these. Wallensten et al. [3] underline the importance of working with prevention in stress-related conditions like ED to reduce the risk of relapse.

To our knowledge, only three studies [30–32] have explored the process of returning to work after sick leave due to ED or work-related strain. Two studies found that facilitating factors and barriers for RTW were both external and internal [30, 31]. Internal facilitating factors were adaptive coping skills and insights about one’s personality and behaviors [30] as well as faith in one’s competence [31, 32] and engagement in meaningful activities [31]. An external facilitating factor found in all three studies was social support [30–32]. Barriers found were external factors such as heavy workloads, conflicts, low influence and lack of structure and clarity at work [31]. However, despite feeling supported, the participants in one study [32] expressed worries about how to handle setbacks and challenges in the future. Altogether, this calls for more research exploring the long-term process of RTW after sick leave due to ED.

In summary, research about RTW for this population is inconclusive. Furthermore, qualitative research focusing on the experience of the RTW process in individuals with ED is limited, especially with individuals who have undergone treatment and returned to work. Consequently, more knowledge is needed on how to better support individuals with ED in their process of a sustainable RTW, and to prevent relapse in sick leave in the future. Therefore, the aim of this study was to explore how individuals who had returned to work after sick leave due to ED experienced the process of returning to work. Specifically, we aimed to identify facilitating factors, significant challenges and needs of further support. This will add to the current understanding of what constitutes a successful RTW, which could be useful for developing better support systems for these individuals.

## Method

### Design and philosophical framework

The aim of this study was to explore experiences of returning to work in individuals previously on sick leave due to exhaustion disorder. For this purpose, a qualitative method with semi-structured interviews was used. The study is grounded in the philosophical framework of critical realism [33], which acknowledge an objective reality while at the same time recognizing that our knowledge of it is inevitably subjective and interpreted. The study was reported in accordance with the Consolidated Criteria for Reporting Qualitative Research (COREQ) guidelines [34].

### Participants and recruitment

Participants were recruited in collaboration with three clinics in Stockholm specialized in rehabilitation for patients with ED [PBM Sweden AB and Capio Hjärnhälsan AB]. The rehabilitation program in all three clinics lasted for 12 weeks and consisted of a multimodal intervention based on CBT with elements of Acceptance and Commitment Therapy (ACT). Before treatment, the individuals had to undergo a multimodal assessment with a licensed psychologist, a licensed physician and either a licensed physiotherapist or a licensed occupational therapist. Inclusion criteria for the rehabilitation included fulfilling the ICD 10th diagnostic criteria of ED [F43.8 A] and being motivated for participating in the group-based intervention. Exclusion criteria were severe depression, moderate/high suicidality, untreated posttraumatic stress or fulfilling criteria for other psychiatric diagnoses assessed as the primary impairment. The inclusion criteria for the current study consisted of having previously been diagnosed with ED and having undergone a rehabilitation program at one of the three clinics. Since the current study was interested in experiences of returning to work after a period of sick leave, participants also needed to have been on sick leave for at least 50% during the rehabilitation period, and, at the time of recruitment, be working at least 50%.

Participants were recruited and interviewed in two phases. Participants from the first and second clinic were recruited during September 2022. The majority of them were interviewed during October and November 2022 and a few in January 2023, due to pragmatic reasons. Since the first two clinics were part of the same health care provider, the research group decided to recruit participants from a third, separate clinic. Inclusion criteria, exclusion criteria and treatment procedure were equivalent for all three clinics. The participants from the third clinic were recruited and interviewed in January 2023.

From clinics one and two, all individuals that had completed the treatment in the last three months and that met the inclusion criteria (*n* = 19) were sent a letter with information about the study. They were also informed that they would to be contacted by phone approximately one week later. Upon calling, two declined to participate due to lack of time or energy and five could not be reached. In total, 12 participants, seven women and five men, agreed to take part in the study. The participants were sent an informed consent form to fill out and return, which was done by 11 participants. However, one participant took part in an interview after giving audio recorded oral informed consent, but did not return the written informed consent form and was therefore excluded from the study. From the third clinic, participants were purposively selected, to better mirror the population as a whole in regards of gender and employment. Eight participants were sent an information letter. When contacted, four participants agreed to participate and filled out the informed consent form, two declined due to lack of time or energy and two could not be reached. In total, 15 individuals were included in the study, 11 from clinics one and two and four from clinic three. Since empirical data [35] has shown that 9–17 interviews are enough to reach saturation, 15 interviews were deemed appropriate for this study. No compensation for participating was offered. Participant characteristics can be seen in Table 1.

**Table 1:** Participant characteristics

	Participants (*n* = 15)
Age (years)	
Mean (sd)	40.9 (8.28)
Gender: n	
Women	10
Men	5
Working with: n	
Human services	3
Media	2
Administration	2
Law, finance and business development	3
Engineering	2
Self-employed/Freelance	3
Percentage of Full-Time Equivalent (FTE) employment	
Mean (sd)	82.9 (17.9)*

* One of the participants did not have a formal degree of employment and could not estimate the working time

### Data collection

The interviews were conducted via video link or telephone between October 2022 and February 2023. For pragmatic reasons, four interviewers were involved: SF conducted four interviews, AN and HH conducted two together and then four interviews each and AR conducted two interviews. SF (female), and AR (male) are licensed clinical psychologists with previous experience of working with people with ED. AN and HH are two female clinical psychology students writing their master’s thesis, with no previous experience of treating patients with ED. The participants did not get any further information about the interviewers. The interviews lasted between 38 and 95 min and the variation in interview duration was mainly due to how much participants elaborated on their responses. When interviews took longer than one hour, the participants were offered a break. All interviews were audio recorded and then transcribed verbatim by the interviewer, or in the case of the interviews conducted by AR, by SF. An interview guide was developed by AR and discussed by all interviewers together, both before and between the interviews (the interview guide can be found under supplementary material). The questions were open-ended and concerned the participants’ experiences of becoming ill, of undergoing rehabilitation as well as their current working situation and their process of returning to work. No repeat interviews were conducted and the transcripts were not returned to participants for comment.

### Ethical statement

This study was approved by the Swedish Ethical Review Authority: 2022-00892-01. All participants received both written and verbal information and had the opportunity to ask questions before agreeing to participate. The recorded interview and transcripts were pseudonymized, e.g., abcd1234, and stored electronically on a secure online platform managed by Uppsala University. Only AR and MB have access to the data. A code key connecting the pseudonyms and personal information was stored in a locked safe by MB. The participants were informed about how the data would be handled, stored and presented.

### Data analysis

The data was analyzed using conventional qualitative content analysis [36]. Conventional qualitative content analysis is an inductive approach, which is suitable when knowledge in a field is limited [36]. The analysis was conducted based on the procedure and concepts presented by Graneheim and Lundman [37] and the analysis focused on the manifest content. According to this procedure, the first step is to select the overall unit of analysis, which in this case was the transcripts of the 15 interviews. The next step is condensing: to divide the text into meaning units, and eventually condensed meaning units, and thereafter label these into codes. The codes are then sorted into themes and sub-themes or categories and subcategories, while bearing the whole context in mind. The main part of the analysis was conducted by the main author SF with assistance of FJ. Initially, all transcripts were read through thoroughly by SF and FJ and notes were taken. Then, relevant units of analysis in each interview were selected. This was done by SF and FJ together for three interviews and then by either SF or FJ for the rest of the transcripts. The coding was conducted by SF and FJ under the supervision of AR. SF and FJ coded one interview together, discussing each code and reaching consensus. SF and FJ then coded half of the remaining interviews each. The further analysis was then conducted by SF under the supervision of AR and the results were continuously discussed in the research group. SF, FJ, JCvdL, MB and AR are all licensed clinical psychologists with previous experience of working with people with ED. SF and JCvdL were working part time at clinic 1 and 2 and FJ at clinic 3, but none of the researchers had been involved in the treatment of the participants included in this study. The coding and analysis was conducted using Microsoft^®^ Excel för Mac Version 16.94. Since the researchers aimed to stay as true to the participants own words as possible, most of the codes were still extensive when the first round of coding was completed. The process of condensing then continued by labeling each code with one or more subjects. Subjects were then gathered together into themes, which were refined throughout the process. The interviews were read again in their entirety to further develop the themes as well as to make sure the themes represented the data [38]. The analysis yielded main themes on a descriptive level [39]. The subthemes however, vary in their level of abstraction and interpretation: some are subthemes on a descriptive level with a slightly higher amount of interpretation and some are instead subcategories, which are more categorical and closer to the participants own phrasing. This mixed level of abstraction is partly due to the aim of the study, where both the overall experiences as well as concrete facilitating and aggravating factors were of interest. Feedback on the results were not obtained from the participants. Example of the coding process can be seen in Table 2.

**Table 2:** Example of the coding process

Quote	Meaning unit	Code	Subject/s	Subtheme	Theme
I don’t function like I used to, and it’s hard for me to learn new things, grasp, and sort information. I used to be really quick, but now I’m careful and cautious in everything I do.	I don’t function like I used to	Doesn’t function as before	Function Change	Coping with lower levels of functioning	Struggling to adapt sustainably to change

Finally, several decisions were made to ensure the study’s trustworthiness. A dense description of the procedure and decisions has been provided, for transferability and dependability [37]. Quotes were used to illustrate how themes represent data and the condensing process was illustrated, which strengthens the credibility of the study [37]. There was a back-and-forth discussion in the research group during the analysis phase, which, together with the use of multiple coders, strengthens the study’s confirmability and credibility [37]. Furthermore, for dependability, the interview guide was made available.

## Results

**Fig. 1:**
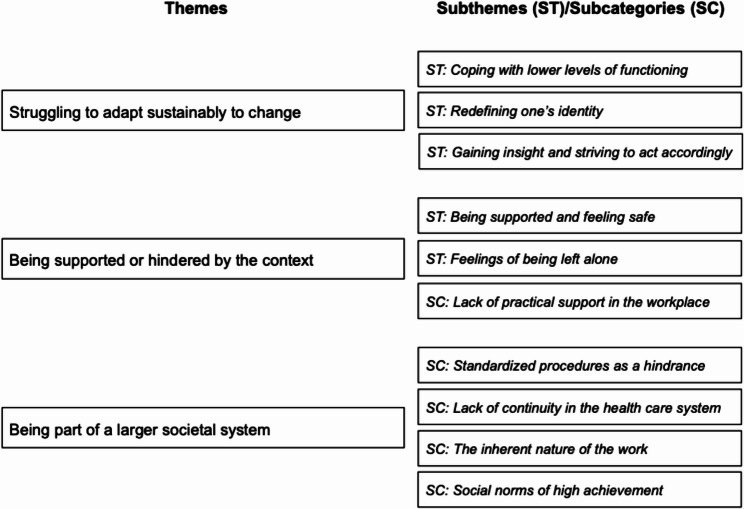
Themes and subthemes/subcategories

The qualitative content analysis yielded three themes: *Struggling to adapt sustainably to change*, *Being supported or hindered by the context* and *Being part of a larger societal system.* The themes and the ten subthemes/subcategories are shown in Fig. 1.

### Struggling to adapt sustainably to change

Suffering from ED presented the participants with new challenges to cope with. This led to a transitional process, where awareness of their own behaviors and needs played a vital role in order for them to act in a more sustainable way that reduces the risk of relapse. The first subtheme, *coping with lower levels of functioning*, reflects that the great majority of the participants described a lowered and more uneven level of functioning, primarily in terms of a lack of physical and mental energy and cognitive impairments. This, in turn, had an impact on their ability to make decisions, plan activities and prioritize. Several of the participants described the reduced level of function as frightening. Furthermore, to evaluate the limits of their functional capacity and to deal with increasing demands constructively were mentioned as challenges when returning to work.

*I think the biggest challenge is finding ways to adapt*,* okey*,* but how do I do this now*,* what is my level of functioning and how do I adapt to this? (Participant 2)*

Furthermore, the great majority of the participants reflected on how suffering from ED had affected their perceived identity, which is reflected in the subtheme *redefining one’s identity*. Notions of being a high achiever and being ambitious were described by many participants as closely linked to their identity, and having to return to work while not functioning as they used to was challenging. Previous conceptions about who they were or who they would like to be was discussed by some as a barrier when trying to adapt to their new circumstances.

During their sick leave and RTW, the participants found themselves in a process of redefining their identity. Several participants described that they did not think they would ever be the same after having suffered from ED. Some of them mentioned having accepted their situation while some felt frustrated. Many participants also discussed relapsing into old habits as a risk factor for relapse in sick leave and mentioned that their old habits were often closely linked with their identity.

…*it has been really difficult for me to realize that I will never be the same person I was before the sick leave […] I was always striving to […] come back and be the same person as before […] and it took even more effort than before to keep up that façade*,* so that was pretty much the reason that I went on sick leave again. (Participant 15)*

The contents of the subtheme *gaining insight and striving to act accordingly* was discussed by the majority of the participants as essential for recovery. It was important for participants to understand themselves, their own behaviors and needs and why they previously had acted the way they had:

…*becoming more aware of behaviors*,* how do I act? Why do I act as I do and how can I kind of think differently or do differently? And that*,* I think*,* was really important. (Participant 7)*

Reflecting on their values helped them do things differently. Novel behaviors differed between participants, but frequently mentioned examples were healthy routines, challenging oneself and to better communicate one’s needs. Being aware in the moment and move more slowly when not in a hurry was also discussed as a helpful new habit. Awareness and acting consciously were described by the participants as key factors for maintaining their new behaviors. A majority of the participants mentioned that behavioral change takes time and that they, because of this, believed that it would be important to have access to long-term support during the whole RTW process.

### Being supported or hindered by the context

While struggling to adapt sustainably to their new conditions, the participants were also in constant interaction with their context. Perceived support, or lack thereof, had a substantial impact on how the great majority of the participants experienced the RTW process. The subtheme *being supported and feeling safe* reflects how support from the context could facilitate the process of returning to work. Both the employer and the health care professionals as well as family and friends have been mentioned as important providers of support. Feeling understood, respected and listened to, created an experience of being safe.

*I got tons of support from family and friends that understood*,* and the employer has been great all the way and has given me space to be in peace […] so I was really*,* really lucky. (Participant 4)*

The support discussed is also of a more practical and structural nature, including help with daily household chores, receiving suggestions of how to adjust work tasks to current levels of functioning, having continuous follow up-meetings at work, and to receive help with prioritizing among tasks.

*I think the key is to have recurrent meetings with my bosses and those I’m working closely with […] I have daily meetings with that person so that I can hand over the task at the end of the day. (Participant 12)*

To be challenged, encouraged and reminded by other people were also discussed as factors facilitating behavioral change as a part of a sustainable RTW process. The participants also found it helpful when they got assistance from others to adapt the level of activity to one’s needs and current functional level. On the other hand, many participants expressed that lack of support led to *feelings of being left alone*, which constitutes the second subtheme. Some of them described being deserted with responsibilities regarding the RTW process, such as having to decide by themselves when to increase the number of working hours or coming up with suitable ways to adapt the work tasks. The feeling of being left alone was discussed in relation to both health care professionals as well as the employer and the workplace:

*When I’m at the primary healthcare center they always ask me “are you going to increase your working time now? Do you want to stop being on sick leave?” You know*,* it’s like it’s all up to me to decide. (Participant 4)*

…*I was left alone and had to plan it [the return-to-work plan] more or less by myself because my boss was very clear that he wanted nothing to do with me so […] my boss wasn’t involved at all. (Participant 13)*

Lack of knowledge regarding ED among both health care professionals as well as employers was discussed as a contributing factor to the feeling of being left alone. Other aspects of this included a general lack of support, not being acknowledged by people around them, or being pressured to return to work before they felt ready. Several participants also mentioned that their symptoms and limited level of function were invisible to the outside world, which made it more difficult for people to understand them. However, it is worth mentioning that a few participants were also satisfied with having the possibility to decide for themselves, since this was associated with experiences of being in control.

The third subcategory, *lack of practical support in the workplace*, was discussed by the majority of the participants as an obstacle to a sustainable RTW process, and as a contributing factor to feelings of being left alone. Managerial deficiencies included absent rehabilitation routines, such as follow-up meetings, and a lack of help in prioritizing work tasks. However, broader deficiencies such as unclear job descriptions, ambiguous demands, withholding information as well as inadequate or insufficient conflict management were also mentioned.

*I wished it was clearer what I was supposed to do […] but it was as if there was no […] job description for me. (Participant 13)*

### Being part of a larger societal system

The majority of the participants also described challenges related to being part of a larger societal system. They had to navigate bureaucratic procedures while at the same time be dealing with societal norms. However, there was a large variation in the participants’ experiences. Some referred to all of the factors within this theme as aggravating, some mentioned one or several as frustrating, while a few did not mention this at all. Hence, the individual participants’ unique situations seem to have greatly affected the way in which participants experienced their relation and/or belonging to a larger societal system.

The subcategory *standardized procedures as a hindrance* highlights that fixed procedures or rules can significantly impact the experience of returning to work. The Swedish Social Insurance Agency has standardized procedures for returning to work that sometimes limit the possibilities of adapting the RTW process to individual needs. This might, for example, be rules about the distribution of working hours during a week or the fixed amount of time on different levels of sick leave. This led to frustration, tiredness, anxiety, or increased stress for many of the participants.

*I can’t work half a day*,* but I can work one day […] a week. But if you have worked for a full day*,* you are considered to be recovered […] I can work 50% spread out over a month*,* but I can’t work 50% of a day and that doesn’t work according to their system. (Participant 14)*

Many participants discussed the impact of timing on the RTW experience, such as when the RTW was initiated or if the RTW happened to coincide with a planned vacation etc. However, matching the RTW or treatment to the individual’s needs was sometimes not possible, due to standardized procedures. A consequence that several participants discussed was the discrepancy between the number of working hours reported and actual hours worked; some of them did not have the capacity to work the hours outlined by the Swedish Social Insurance Agency.

Many participants also mentioned a *lack of continuity in the health care system*, which constitutes the second subcategory. They described meeting different health care providers in different phases of their sick leave and treatment, and that it was exhausting having to tell their story over and over again and repeatedly trying to make themselves heard. This was perceived as especially demanding given their reduced level of functioning. One participant reflects on finishing the rehabilitation program:

*At least for me it was really hard*,* since I had no doctor to go back to that had made the referral in the first place. I had to go back to the general practitioner and talk about what I had talked about for three months and then start over with a new rehabilitation plan. (Participant 15)*

The third subcategory, *the inherent nature of the work*, was discussed by many participants as another barrier for a sustainable RTW process. The participants mentioned that their job entails working conditions that make sustainable adaptations, such as adjusting the pace of work or concretizing tasks, difficult or even impossible. The forms of obstacles varied between participants. Conditions mentioned were project-driven jobs, jobs that involved strict deadlines or obligations to report to higher authorities, or mutual dependency on colleagues for effective work flow and project progression. Another example was when a job entailed working with other people’s needs, such as health care professionals and social workers.

… *I guess that would be the nature of the work*,* that there is no possibility for adaptations in the way that I would have needed […] I can’t work only with administration […] It’s not possible to remove the tasks that are hard for me. (Participant 2)*

Many of the participants also mentioned *social norms of high achievement*, which constitutes the fourth subcategory. These norms could either be found in the workplace, in the professional field where the participants were engaged or was discussed as a societal phenomenon.

*It’s very competitive […] you are expected to quickly advance through career stages. You are working with high performers at high speed […] things like this has been challenging for me during my working life. (Participant 12)*

These norms were discussed by the participants as a contributing factor for developing ED, as well as an aggravating circumstance when returning to work after sick leave.

## Discussion

This study aimed to explore how individuals who have returned to work experienced the process of returning to work after sick leave due to ED, and to identify facilitating factors, significant challenges and further support needs. The results of the qualitative content analysis showed that the participants were struggling to adapt sustainably while at the same time being in constant interaction with their context and managing the consequences of being part of a larger society. The present study adds to the knowledge about the RTW process for individuals previously on sick leave due to ED. Compared to previous research [30–32], a great majority of the participants in this sample had been working at least part-time for several months and could therefore contribute with valuable information about the long-term process of RTW.

The three main themes can be seen as representing different levels: the individual level, the group level and the system level. In the present study, both facilitating as well as aggravating factors for returning to work could in some way be found on all levels. In many respects, the results align with previous research on what individuals experience as supportive for recovery after ED [16]. This indicates unsurprisingly that several facilitating as well as aggravating factors are shared between the recovery process and the RTW process. 

On the individual level, gaining insight and striving to act accordingly was a facilitating factor for a successful RTW process, which is in line with the results of Norlund et al., [30]. On the other hand, trying to live up to one’s self image and acting as if the circumstances had not changed was described as a barrier. These results also align with a recent study by Aronsson et al., [39], suggesting the long-term recovery process from ED can be seen as a “long and rocky road”, where the individuals’ own behaviors were both facilitators and barriers to the process. Furthermore, the experience of never being the same person as they previously were, was discussed by the participants in this study as well. The concept of identity is also addressed in another recent study [40] which found that having been diagnosed with ED forced you to revisit and reevaluate your identity. Hence, supporting people to continue being aware about their needs and values, and to maintain new behaviors could be important factors for preventing relapses in sick leave in this population, particularly if these new behaviors deviate from the previous self-image.

The struggle with identity can be linked to the concept of psychological (in)flexibility [41]. Psychological flexibility is the main focus of ACT and refers to the ability to be in contact with oneself and the present moment, acting according to one’s values and being able to alter one’s behavior in a flexible way when the context changes [41]. When looking at the current study’s result from this point of view, one could argue that the participants’ level of psychological flexibility differs, where some express frustration with not being able to live according to the same ambitions as before. Hence, their level of psychological flexibility might have had an effect on how they experienced the RTW. The treatments in all three clinics used for recruitment in the current study included some elements of ACT, but it was not the main component. This suggests that psychological flexibility as defined in ACT could be a valuable variable to target further in treatments, to support a sustainable RTW on an individual level.

The group level includes actors such as the employer and workplace, health care providers, and family and friends. Lack of knowledge and support from these actors led to feelings of being left alone, which was expressed as a barrier to a successful RTW. These results are in line with a recent study exploring physicians’ experiences of assessing patients with fatigue [42]. The physicians in the study reported frustration and difficulties in assessing, treating and supporting patients with fatigue and/or ED, due to lack of time or the complex nature of these conditions, to name to name a few examples. Previous research also discussed lack of communication and coordination between different stakeholders as barriers to RTW and in general, lack of support has been found to be a common barrier for RTW in several different samples, from common mental disorders to pain conditions [25, 30, 43, 44] Hence, the individual’s context plays an important role in the RTW process. To facilitate this process, increasing knowledge about stress-related disorders and their consequences in health care professionals and employers are of relevance, as well as facilitating good communication between the individual returning to work and their context. This includes clarifying the specific roles and responsibilities of different actors and individuals. Furthermore, since the conditions at the workplace are intertwined with higher levels of organizational leadership, the overall organization has a responsibility to proactively structure the work in a way that enables the more immediate context to support the individual to RTW in a sustainable way.

Within the theme *Being supported or hindered by the context*, participants articulated a wish for experts to take charge and make decisions based on their expertise. Participating in one’s rehabilitation process and being responsible for the process led to increased and unwanted stress and anxiety for several participants. This might be explained by the fact that participants described a lowered level of functioning and therefore did not have the same sense of competence as they previously had. However, some participants did not report the feeling of being left alone, but instead felt more in control by taking charge of their own process. Therefore, in addition to structural shortcomings of different stakeholders, there might also be a possibility that lack of trust in one’s own competence could aggravate the situation. This would be in line with previous research that found self-efficacy as a factor affecting how participants perceived their future and possibilities [25].

Finally, several barriers were found on the level of the larger societal system, while facilitating factors were hardly mentioned at all. The lack of explicit references to such factors could be attributed to the way the larger societal system in fact is mediated through the context – such as employers and health care workers. When the societal system is well functioning and matches the individual’s needs, facilitating factors thus become invisible. The perceived flaws of the system become apparent when the context’s possibility of facilitating for the individual is hampered – such as when *standardized procedures* regulate actors in the context in such a way as to render them unable to provide the support needed by participants.

Further, exposure to high achievement norms was experienced as a barrier to a sustainable RTW. This is in line with Hörberg et al. [45] who found spending some time free from demands as a crucial factor for wellbeing after having experienced stress-related illness. How these norms of achievement appear, however, differs in different settings. Nevertheless, regardless of setting, to lower expectations in relation to achievement and communicate this the with the individual is a factor for employers to consider in order to facilitate the RTW process. Again, this issue is a responsibility for the employer and workplace as well as the larger organization. Altogether, these results expand the knowledge on the RTW process after sick leave due to ED and the results from the current study are in line with previous research on similar populations [25, 30–32].

### Strengths and limitations

The study has several strengths as well as limitations that should be considered. Firstly, three different clinics from two different health care providers were contacted for recruitment, to make the results less dependent on specific routines or procedures at one clinic. The clinics were all specialized in assessing and treating ED and there was a variation in age, gender and occupation among participants. To make the sample more representative of the population, only women were recruited from the third clinic. Altogether, these decisions can be seen to strengthen the study’s credibility [46]. However, few of the participants in the final sample were working in fields commonly associated with ED, such as health care and education [47]. This is a limitation, since the experiences in the current sample therefore might differ somewhat from the experiences of people working in those fields, where common challenges are understaffing and high emotional and psychological strain. Moreover, the use of several interviewers could be seen both as a strength and a limitation. This increases the risk of having interviews conducted differently, but it can also contribute to more nuances and variation. An interview guide was used to facilitate a shared approach.

Finally, a challenge when creating themes and subthemes/subcategories was mutual exclusiveness [37]. Several of the subthemes can be seen as parts of the same concept, as well as interacting with each other. The second theme in this study, *Being supported or hindered by the context*, is an example of this. The third subcategory, *Lack of practical support in the workplace*, is closely related to the second subtheme *Feelings of being left alone.* Both are concerned with lack of support, one more emotionally and one more practically. These concepts are somewhat intertwined but were here chosen to be separated to emphasize the impact of organizational deficiencies on RTW. In a similar vein, the division between group level and system level is a simplification that does not capture the complexity of these domains, since they intersect and are in constant interaction.

When conducting an inductive qualitative content analysis, the researchers need to reflect on their pre-understanding of the subject of analysis [48]. For the current study, the main researchers were experienced in assessing and treating individuals with ED, which could be a risk regarding confirmation biases, among other things [49]. To counter this tendency, the main author has throughout the process engaged in reflexivity [46]. However, a pre-understanding can also strengthen the study, as an active and conscious use of the researchers pre-understanding can bring value to several parts of the research process and play a necessary part in knowledge development [49].

## Conclusion

In conclusion, both individual factors, the surrounding context and the larger societal system have an impact on individuals’ experience of returning to work after sick leave due to ED. This emphasizes the well-known complexity of the RTW process and the multi-leveled nature of factors involved in determining the outcome. Overall, the results of this study suggest the need for further and, in particular, prolonged support for these individuals even after they have undergone clinical treatment. In a clinical context, such support may include programs designed to facilitate behavioral maintenance and realization of insights gained from the main clinical treatments of ED, for instance concerning the long-term renegotiation of one’s values, or enhancing one’s psychological flexibility to adapt sustainably to changing capacities as these are further transformed during RTW. Further, lack of both emotional and practical support, particularly in relation to the workplace, calls for enhanced external support for these individuals. Some ways to achieve this could be to enhance stakeholders’ understanding of stress-related illnesses, to foster effective communication about needs and circumstances – particularly between the employer and employee – and to clearly define roles and responsibilities in the workplace during the process of RTW. Further, employers and the associated organizations need to work proactively to ensure that the structure and routines of the work enables the support needed for returning to work in a sustainable way. This study offers valuable insights into the experiences of returning to work, as well as aggravating and facilitating factors of the RTW process after sick leave due to ED. These insights could be useful when further developing support systems for individuals undergoing such a process. Future research should focus on how this prolonged support can be delivered, both in terms of individual interventions and interventions on an organizational level.

### Reflexivity statement

SF, FJ, JCvdL, MB, and AR are all licensed clinical psychologists with previous experience of working with people with ED. AN and HH are two clinical psychology students writing their master’s thesis. SF and JCvdL were working part time at clinic 1 and 2 and FJ at clinic 3, and they were therefore well informed of the treatment procedure at the different clinics. None of the researchers have been involved in the treatment of the participants included in this study.

## Supplementary Information

Supplementary Material 1.

## Data Availability

The interview guide used in the study can be found under supplementary materials. The data analyzed during the current study are not publicly available due to its sensitive nature and to protect participants confidentiality. Requests regarding any data should be sent to the corresponding author.

## Abbreviations

ACT: Acceptance and commitment therapy
CBT: Cognitive behavior therapy
CMD: Common mental disorders
COREQ: Consolidated Criteria for Reporting Qualitative Research
ED: Exhaustion Disorder
ICD: International Statistical Classification of Diseases and Related Health Problems
RTW: Return to work
