# Supplementary material for: Experiences of returning to work after sick leave due to exhaustion disorder: a qualitative content analysis
Source: BMC Psychol. 2025 Dec 20;14:117. doi: 10.1186/s40359-025-03873-9 (PMC12849070; doi:10.1186/s40359-025-03873-9)
Supplement: Supplementary file 1 — Supplementary Material 1. [file 40359_2025_3873_MOESM1_ESM.docx]

**Interview Guide for the Research Study:***Experiences of returning to work after sick leave due to Exhaustion Disorder: a qualitative content analysis*

**Background**

- Tell me a little about yourself.
  - Family situation.
  - Hobbies.
  - Education.

**Sick Leave**

- Tell me about your sick leave for Exhaustion Disorder.
- When were you placed on sick leave?
- How long were you on sick leave before starting your rehabilitation?
- How did you perceive the process of your sick leave (e.g., contact with the prescribing doctor and the Social Insurance Agency)?
- What are your views on the diagnosis of Exhaustion Disorder (e.g., did it align well with your problems or did you feel something was missing)?
- What do you think contributed to your Exhaustion Disorder?
- What support did you receive from others during and in connection with your sick leave; employer, colleagues, family, and friends?

**Rehabilitation**

- Tell me about the rehabilitation you underwent.
- What was the rehabilitation about?
- How did you experience the treatment and support?
- How did you perceive the outcome?
- What was particularly good about the rehabilitation? Please elaborate.
- Was there anything you missed in the rehabilitation? Please elaborate.

**Work**

- What is your current employment?
- How much do you work (e.g., part-time)?
- How long have you been working there?
- Is it the same job you had when you went on sick leave?
- Briefly describe a typical workday.
- How do you enjoy your work?
- Are there routines for managing mental health issues? Please elaborate.
- Do you know where to turn if you need support? Please elaborate.

**Return to Work**

- Tell me about your experiences of returning to work after your sick leave.
- Was a plan developed for your return to work? Please elaborate.
- How did you experience the transition from being on sick leave to working again?
- What was helpful from the rehabilitation at this stage (e.g., strategies)?
- What hindered or created difficulties at this stage?
- Tell me about the support you received from others during your return to work; that is, employers, colleagues, family, and friends.
- Did you miss anything in your return to work (e.g., contact with a psychologist)?

**Support Program**

- What are your views on the need for support after sick leave and rehabilitation?
- Would you have wanted additional help after rehabilitation? Please elaborate.
- If you could design a support program for people returning to work after Exhaustion Disorder, what would you like it to include?
- What are your thoughts on the possibility of receiving this type of support remotely (e.g., via the internet)?
- How long should a support program be provided after returning to work? Please elaborate.
- Many people with mental health issues go on sick leave again within three years after returning to work. Why do you think this happens?
- What would you like to see more of to prevent new sick leave; from society, employers, or colleagues?

**In Conclusion**

- Is there anything else you would like to add? Please elaborate.
- Do you have any questions about the interview or the research study? Please elaborate.
- Once the study is completed, would you like to receive the results?
